# Injectable Biomimetic Hydrogel Constructs for Cell-Based Menopausal Hormone Therapy with Reduced Breast Cancer Potential

**DOI:** 10.34133/bmr.0054

**Published:** 2024-08-09

**Authors:** Chungmo Yang, Heeseon Yang, Hyerim Kim, Nanum Chung, Jungwoo Shin, Hyewon Min, Kangwon Lee, Jung Ryeol Lee

**Affiliations:** ^1^ Department of Obstetrics and Gynecology, Seoul National University Bundang Hospital, Seongnam 13620, Republic of Korea.; ^2^ Program in Nanoscience and Technology, Graduate School of Convergence Science and Technology, Seoul National University, Seoul 08826, Republic of Korea.; ^3^ Department of Translational Medicine, College of Medicine, Seoul National University, Seoul 03080, Republic of Korea.; ^4^ Department of Applied Bioengineering, Graduate School of Convergence Science and Technology, Seoul National University, Seoul 08826, Republic of Korea.; ^5^ Research Institute for Convergence Science, Seoul National University, Seoul 08826, Republic of Korea.; ^6^ Department of Obstetrics and Gynecology, Seoul National University College of Medicine, Seoul 03080, Republic of Korea.

## Abstract

Hormone replacement therapy (HRT) has been a primary method in menopausal women and patients with ablated ovaries, but safety has been a concern. Cell-based HRT has emerged as an alternative approach without side effects causing pharmaceutical HRT via 3-dimensionally engineered constructs layering ovarian hormone-producing cells. In this study, we applied micro-sized ovarian cell-laden hydrogel beads as an approach to cell-based HRT using a minimally invasive method in the menopausal rat model. Here, we constructed GC/TC-laden microbeads (GTBs; GC, granulosa cell; TC, theca cell) that allow crosstalk between endocrine cells, encapsulating multiple beads for the figuration of the original ovary. We assessed the ovarian hormone production function of GTB through in vitro culture for 90 days. We applied it to a menopausal rat model and confirmed that GTB-injected rats restored their endocrine function, leading to the regeneration of the thinned endometrium and the maintenance of regular estrous cycles in some individuals. Additionally, it was observed to alleviate menopausal symptoms, including body weight gain and osteoporosis. Notably, the GTB-injected rats did not show mammary gland hyperplasia observed in the pharmaceutical HRT groups and exhibited fewer p53- and KI67-positive and an increase in phosphatase and tensin homolog-positive mammary gland epithelial cells compared to pharmaceutical hormone-treated rats. These results suggest that GTB-based HRT could present a lower risk of breast cancer compared to conventional pharmaceutical-HRT use. Our study highlights the potential of cell-based HRT using an injectable artificial ovary, offering a safer alternative for women requiring HRT.

## Introduction

Women undergo metabolism regulated by ovarian functions for almost their entire life. However, as women age, they eventually lose their ovarian functions due to repeated menstrual cycles, leading to menopause. Menopause means the end of the menstrual cycle with decreased ovarian follicles capable of hormone production. After menopause, the hormone balance collapses and causes side effects such as loss of menstrual cycle, vasomotor symptoms, urogenital atrophy, cardiovascular disease, osteoporosis, mood swings, and sleep changes [1]. As the symptoms mainly occur from a deficiency of the ovarian sex steroid hormone, hormone replacement therapy (HRT) is widely applied to treat menopausal-related diseases. However, HRT can cause prevalent side effects, such as breast cancer or stroke [2–4]. Breast cancer is a common side effect of conventional HRT, and it has been reported that the risk increases when using combined estrogen/progesterone regimens [3,5]. The combination of progesterone with estrogen is used to prevent the occurrence of endometrial carcinoma in women with an intact uterus [6]. However, estrogen and progesterone induce the proliferation of hormone receptor-positive breast cancer cells compared to estrogen alone [7].

There is an alternative emerging principle, “cell-based HRT”, as a tissue engineering and regenerative medicine approach [8]. Sex hormones (such as estrogen, progesterone, activin, and inhibin) are produced mainly in the gonad (ovary in the case of women), and they regulate gonadotropin-releasing hormone (GnRH) released from the hypothalamus and the secretion of gonadotropins from the pituitary gland via negative and positive feedback mechanisms [9]. The hypothalamus–pituitary–ovary (HPO) axis plays a crucial role in maintaining female endocrine function, regular menstrual cycle, and fertility. After menopause or decreased ovarian functions caused by primary ovarian insufficiency and related diseases, most of the side effects are from hormone deficiency produced by gonads, leading to disruption of the HPO axis system [10,11]. The cell-based HRT aims to generate ovarian sex hormones from the ovary-originated cells such as granulosa cells (GCs) and theca cells (TCs). Such reports have provided proof that bioartificial constructs exhibited hormone production and prevented menopausal symptoms by sustaining hormonal concentrations at physiological levels [8,12]. For example, the alginate-based constructs were prepared to produce ovarian sex hormones with layered GCs and TCs [13]. It was also revealed that the layered constructs are more efficient than mixed co-encapsulation, which is important in biomimetic design. The constructs exhibited hormone production of a normal range after ovariectomy, which prevented osteoporosis in long-term experiments. Later, the authors revealed that stem cells can promote the bioartificial constructs to enable stable and prolonged hormone secretion [14].

In recent years, the field of 3-dimensional (3D) encapsulated cells has gained prominence as a promising approach to treating tissue and organ injuries, offering marked potential for advancing cell-based therapies [15,16]. Hydrogel, a versatile biomaterial, has emerged as a preferred matrix for encapsulating various types of cells, including mesenchymal stem cells (MSCs), endothelial cells, osteoblasts, and even cancer cells. Multiple encapsulation techniques, such as micro-molding, electrostatic extrusion, microfluidic devices, and bioprinting, have been explored [17–19]. The simplicity and reliability of encapsulating cells within hydrogel beads make this method a widely adopted choice. These microspheres offer versatility in administration, including transplantation, direct tissue injection, and intravenous delivery [20–22]. The researchers have demonstrated efficacy in diverse applications, ranging from bone and cartilage regeneration to neovascularization promotion and targeted cancer therapy. For instance, hydrogel beads were developed for bone/cartilage regeneration, neovascularization, and cancer therapy [23].

In cell-based HRT, the alginate beads were designed to encapsulate GCs and TCs, mimicking the structural organization found within the native ovarian follicles [13]. The previous research concluded that layered structural beads are more efficient than structures of hydrogel beads in producing hormones continuously. This structural fidelity to the original ovarian follicles fosters a dynamic interaction between GCs and TCs, closely resembling the natural hormonal interplay within the ovary. The inherent harmony achieved through structural mimicry not only enhances hormone production but also paves the way for an alternative native ovary-like environment. This environment holds the promise of providing more effective treatments for conditions stemming from decreased ovarian function, thus offering renewed hope and improved quality of life for women facing these challenges [24]. Furthermore, this innovative approach not only potentially mitigates menopausal symptoms but also may have broader implications for enhancing women’s reproductive health and well-being throughout their lives.

Herein, we introduce a minimally invasive method by injecting micro-sized cell-laden hydrogel beads. The micro-sized hydrogel beads were designed to mimic ovarian structures, such as the distribution of ovarian follicles within the ovary. The micro-sized beads can be prepared using the electrostatic extrusion method with isolated GCs and TCs. The GC and TC beads were sized about 200 μm and 800 μm, respectively. The hydrogel beads were injected into the subcutis of ovariectomized (OVX) rats with hyaluronic acid (HA) hydrogel.

## Materials and Methods

### Ovarian cell isolation and culture

All animal protocols and procedures in this study were approved by the Institutional Animal Care and Use Committee of Seoul National University Bundang Hospital (approval number: BA-2007-299-063-01). All rats were bred and housed in a controlled setting under specific pathogen-free conditions. The conditions included regulated temperature and humidity, a 12-h cycle of light and darkness, and they received food and water ad libitum.

As an ovarian endocrine cell donor for fabrication of GC/TC-laden microbeads (GTBs), 21-day-old immature female Sprague–Dawley rats were subcutaneously injected with 17β-estradiol (E_2_; Sigma-Aldrich, St. Louis, MO, USA) dissolved in cottonseed oil (1.5 mg/0.2 ml) for 3 consecutive days. The ovaries were harvested and then placed in ice-cold HEPES-buffered medium 199 (M199; Gibco, NY, USA) supplemented with 5% fetal bovine serum (FBS; WelGene, Deagu, Korea) and 1% antibiotic-antimycotic (AA; Sigma-Aldrich). After washing with ice-cold M199, the GCs were released by ovary puncturing method with a 31-gauge syringe and then collected by centrifugation (300 × *g*) for 3 min. For TC collection, the remaining ovaries were chopped and then incubated in 0.2 ml/ovary of collagenase-DNase I solution (M199 with 2 g/ml of collagenase, 10 μg/ml of DNase I, and 5% FBS) for 60 min at 37°C. The chopped mixture was washed with McCoy’s 5A medium (Gibco) and seeded. The GCs and TCs were each cultured in a 75T flask and reached 80% confluency. The purified GCs were cultured in McCoy’s 5A medium with 5% FBS, 1% AA, 1% Insulin-Transferrin-Selenium (ITS) +1 (ITS+1; Sigma-Aldrich), 10 nM insulin-like growth factor-I (IGF-I; Sigma-Aldrich), 200 ng/ml follicle-stimulating hormone (FSH; Sigma-Aldrich), 100 nM E_2_, and 10 μM Rock inhibitor (Selleckchem, Houston, TX, USA). Similarly, the TCs were grown in McCoy’s 5A medium with 5% FBS, 1% AA, 1% ITS+1, 10 nM IGF-I, and 100 ng/ml luteinizing hormone (LH; Sigma-Aldrich).

### Characterization of GC and TC

#### Immunofluorescent stain for GCs and TCs

Cultured cells were fixed with 4% paraformaldehyde (PFA) for 15 min at room temperature (RT) and then washed with Dulbecco’s phosphate-buffered saline (DPBS). For permeabilization, 0.5% of Triton X-100 was treated for 10 min at RT. After washing with DPBS, the cells were blocked with 3% bovine serum albumin (BSA) in DPBS for 1 h and then incubated at 4°C overnight with primary antibodies diluted in 0.1% BSA in DPBS. The primary antibodies used in this study are anti-CYP19 (BS-1292R, Bioss, Woburn, MA, USA) and anti-CYP17A1 (BS-54306R, Bioss). Cells were rinsed 3 times in DPBS before incubation for 1 h at RT with the goat anti-rabbit IgG H + L Alexa Fluor 488 secondary antibodies (Invitrogen, Waltham, MA, USA) diluted in 0.1% BSA in DPBS. The nuclei were labeled using 4′,6-diamidino-2-phenylindole (Invitrogen).

#### Flow cytometry

The purity of GCs and TCs was analyzed using flow cytometry with a cell-specific marker. Briefly, fractions of each cell were filtered using a cell strainer (70 μm) and then fixed in 4% PFA. After washing with DPBS, the cells were permeabilized using Triton X-100 (0.5%) and treated with 3% BSA for blocking. Next, cells were incubated with primary antibodies (anti-CYP19 or anti-CYP17A1) at 4°C overnight. Cells were rinsed using flow cytometry buffer (DPBS with 5% FBS and 0.1% sodium azide) and incubated with goat anti-rabbit IgG H + L Alexa Fluor 488 secondary antibodies for 1 h. After washing off the antibody unbounded cells, the cells were resuspended in flow cytometry buffer in the end, and analysis was conducted using a BD FACSCalibur Flow Cytometer (BD Biosciences, CA, USA). Data were analyzed by using FlowJo V10.5.3 software (FlowJo LLC, Ashland, OR, USA).

### Preparation of GTBs

The microbeads were prepared by the electrostatic extrusion method, in which a 9-kV positive voltage was applied to 1.5% alginate solution in Hanks’ balanced salt solution (HBSS; Biowest, Nuaillé, France) via a 28-gauge stainless steel needle with a continuous flow rate of 0.5 ml/h using a syringe pump (NanoNC, Seoul, Korea) for 20 min at RT. The alginate solution, containing 5 × 10^6^ GCs, was dropped from the needle into a 1% calcium chloride (Sigma-Aldrich) solution. The alginate microbeads were collected and washed with distilled water and HBSS. For secondary beads, the washed microbeads were carefully mixed with alginate solution (1.5% in HBSS with 1 × 10^7^ of TCs), suspending TCs. The mixed solution was dropped into a 2% calcium chloride (Sigma-Aldrich) solution via a 20-gauge stainless steel needle applied with 11-kV positive voltage. Then, the second microbeads were collected and washed using the same steps as the previous ones. The fabricated GTBs were carried in a medium for cell study or HA solution (5% w/v) for in vivo injection.

### Cell viability and hormone synthesis function of GTBs in vitro

To assess the viability and hormone synthesis function of ovarian endocrine cells within GTBs, the GTBs were plated on semipermeable transwell inserts (Corning, NY, USA) and cultured for 90 days in the transwell system. Live/dead assay was conducted following the manufacturer’s instructions. Briefly, GTBs were transferred in a 6-well plate and then incubated with a solution of Calcein AM and ethidium homodimer-1 (LIVE/DEAD kit, Invitrogen) for 30 min in darkness. GTB images were obtained by confocal laser scanning microscopy (Carl Zeiss, Oberkochen, Germany). Additionally, to verify the function of hormone production of the ovarian endocrine cells in GTB, E_2_ and progestogen (P_4_) were measured in GTBs spent medium using rat enzyme-linked immunosorbent assay kits (E_2_: KEG014, R&D Systems, Minneapolis, MN, USA; P_4_: CSB-E07282r, CUSABIO, Wuhan, China).

### Bilateral ovariectomy and injection of GTBs

Female Sprague–Dawley rats (8 weeks old) underwent bilateral ovariectomy to remove the ovarian tissues. After 4 to 5 weeks, when serum sex hormone levels reached a stable baseline, the OVX rats had a GTB injection (*n* = 9) or 4 types of pharmaceutical hormone delivery. The GTBs carried in HA solution were transplanted under the neck through subcutaneous injection using a syringe (18-gauge). The GTBs prepared by ovarian endocrine cells harvested from one immature female rat were injected into one OVX rat. For pharmaceutical hormone delivery, E_2_ or/and P_4_ (Sigma-Aldrich) dissolved in cottonseed oil were administered through subcutaneous injection in the same region of GTB injection. Hormone injection was administered at intervals of 4 to 5 days to mimic estrous cycle of rats. These 4 pharmaceutical HRT groups were as follows: low E_2_ (0.1 mg/kg, *n* = 4), low E_2_ + P_4_ (0.1 mg/kg and 2.5 mg/kg, respectively, *n* = 4), high E_2_ (1 mg/kg, *n* = 4), or high E_2_ + P_4_ (1 mg/kg and 2.5 mg/kg, respectively, *n* = 6). The doses were determined based on previous studies [8,12,25]. The effects of HRT were assessed through comparison with the OVX (*n* = 9) and ovary-intact rats (*n* = 9).

### Endocrine function of GTBs in vivo

To verify the recovery of endocrine function in OVX rats receiving HRT, serum samples were collected through the jugular vein once a week in the morning (11:00 AM). The E_2_ and P_4_ were measured using rat enzyme-linked immunosorbent assay kits (E_2_: KEG014, R&D Systems; P_4_: CSB-E07282r, CUSABIO) according to the manufacturer’s protocols. The level of gonadotropins was measured in serum using rat enzyme-linked immunosorbent assay kits (FSH; E-EL-R0391, Elabscience, Houston, TX, USA; LH; E-EL-R0026, Elabscience) according to the manufacturer’s recommendations.

### Uterine weight, histology, and estrous cycle

At 12 weeks after HRT, uterine tissues were harvested from rats, and gross images were obtained. Then, the uterine tissues were fixed in 4% PFA, dehydrated using a graded series of alcohol, embedded in paraffin, and sliced (6 μm thick sections) for hematoxylin and eosin (H&E) staining. The endometrial thickness was analyzed quantitatively using ImageJ software (National Institutes of Health, Bethesda, MD, USA). Specifically, the endometrial thickness of an individual was obtained by dividing the uterus into 3 compartments (top–middle–bottom) and calculating the average endometrial thickness of each compartment.

The estrous cycle was checked daily for 2 weeks, starting at 10 weeks after HRT. Vaginal smears on a microscope slide were stained with H&E and analyzed to identify the 4 stages of the estrous cycle (proestrous, estrous, metestrous, and diestrous) based on the composition of cell types, including epithelial cells, cornified cells, and leukocytes. Estrous cycles recurring every 4 to 5 days were categorized as regular, while individuals exhibiting prolonged estrous or diestrous cycles (each exceeding 5 days) or cycles lasting more than 6 days were considered irregular.

### Osteoporosis assessment

#### Dual-energy X-ray absorptiometry measurement

Once every 2 weeks, fat distribution, whole body bone mineral density (BMD), and bone mineral content (BMC) were analyzed using dual-energy x-ray absorptiometry (DEXA, InAlyzer, MEDIKORS Inc., Seongnam, Gyeonggi, Korea) under anesthesia.

#### Micro-computed tomography imaging

The femoral bone specimens were subjected using a SkyScan 1172 CT scanner (Bruker, Belgium). The computed tomography (CT) scanner was set with parameters including a 13.38-mm image pixel size, 59-kV source energy, 0.5-mm Al filter, 167-mA source current, and a 0.451 rotation step. NRecon (Bruker, Belgium, V1.7.0.4) and micro-CT (μCT; SkyScan, Bruker, Belgium, V1.17.7.2+) were employed for image reconstruction and CT analysis. Various bone morphometric parameters, namely, bone volume fraction (BV/TV), trabecular thickness (Tb.Th), and trabecular separation (Tb.Sp), were analyzed in this study.

### Rick of breast cancer

#### Immunohistochemistry of mammary gland

To confirm the degree of progression to hyperplasia in mammary glands after HRT, mammary glands were harvested from rats, fixed in 4% PFA, embedded in paraffin, and sectioned at a thickness of 6 μm. Following deparaffinization and rehydration, antigen retrieval was performed using 10 mM citrate buffer (pH 6.0 for p53 and KI67, pH 9.0 for phosphatase and tensin homolog [PTEN]). Sections were then treated with peroxidase-blocking solution (DAKO, Carpinteria, CA, USA) and incubated with primary antibodies at 4°C overnight: anti-p53 (ab131442, Abcam, Cambridge, UK), anti-PTEN (ab267787, Abcam), and anti-KI67 (NB500-170, Novus Biologicals, Littleton, CO, USA). After rinsing with PBS, the sections were incubated with horseradish peroxidase-conjugated secondary antibodies. The 3,3′-diaminobenzidine substrate was utilized to visualize the localization of target proteins, and slides were stained with hematoxylin as a counterstain. Images were obtained using a microscope (Nikon, Tokyo, Japan). The number of mammary gland epithelial cells positive for p53, PTEN, and KI67 were counted using ImageJ software.

### Statistical analysis

All data are presented as mean ± standard error of the mean (SEM). Statistical differences were analyzed using Student’s *t* test and one-way analysis of variance followed by Tukey’s multiple comparisons test. All statistical analyses were performed with the GraphPad Prism software, version 10.0 (GraphPad Prism Software Inc., San Diego, CA, USA). A *P* value of less than 0.05 was considered statistically significant. Biological replicates were conducted using separate populations of animals.

## Results

### Cell isolation and characterization

**Fig. 1.**
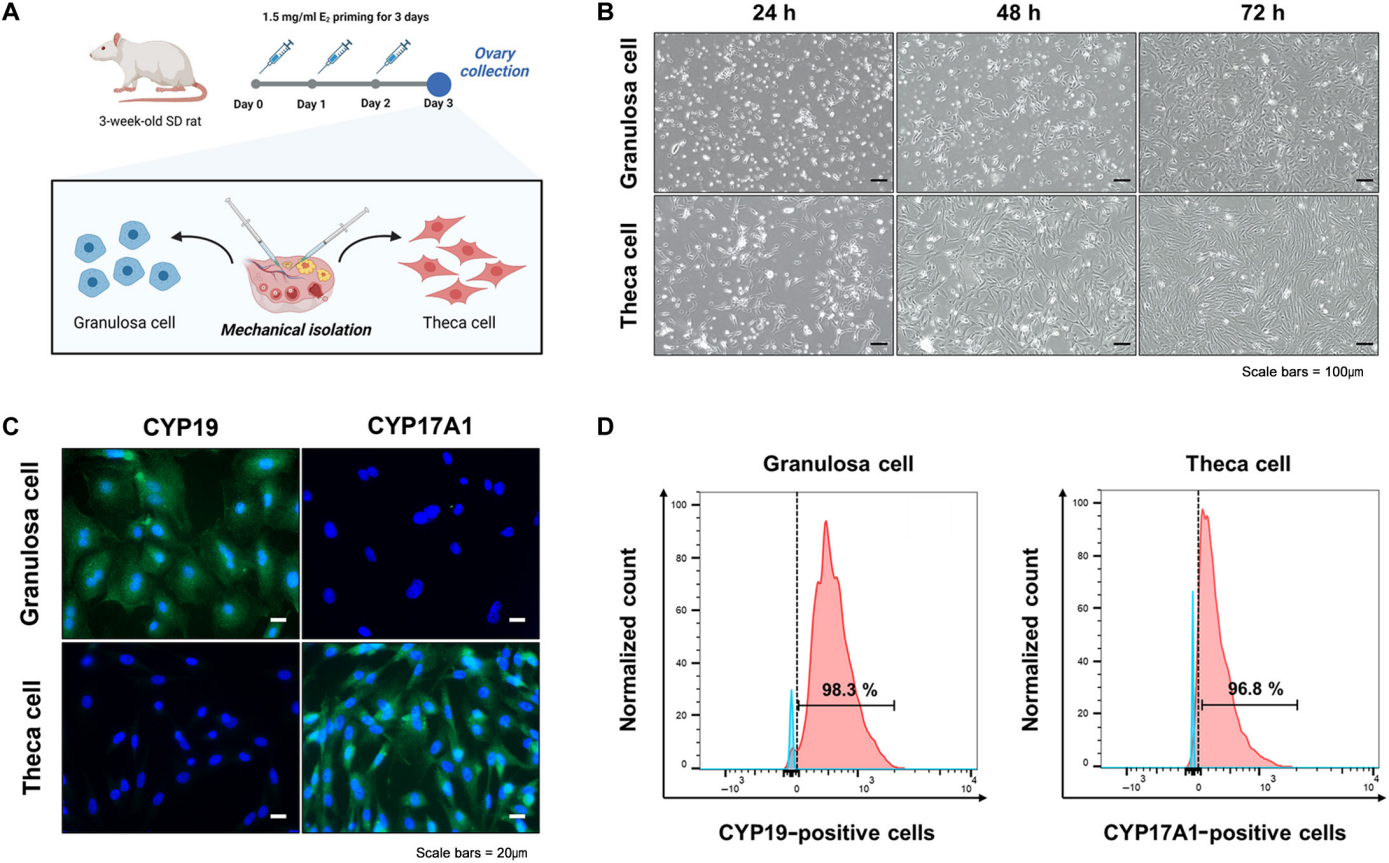
Characterization of mechanically isolated hormone-producing ovarian cells. (A) Hormone-producing ovarian cells are isolated by microdissection from the E_2_-primed ovary of 3-week-old female rats. (B) Morphology of granulosa cells (GCs) and theca cells (TCs) during a 72-h culture. Scale bars, 100 μm. (C) Fluorescence images of the staining of GC and TC with anti-CYP19 granulosa cell marker (green), anti-CYP17A1 theca cell marker (green), and DAPI (blue) nuclear counterstain. Scale bars, 20 μm. (D) The quantitative analysis of cell purity after staining of anti-CYP19 granulosa cell marker and anti-CYP17A1 theca cell marker, respectively, using flow cytometry.

Ovarian endocrine cells were isolated from E_2_-primed rat ovaries using a combination of mechanical puncturing and collagenase-DNase I digestion, as described in Fig. 1A. The cells exhibited morphological differences after 24 h. As reported previously [26,27], GCs exhibit a pebble-like and cuboidal shape, while TCs display fibroblast-like and elongated morphologies. Both cell types maintain these characteristics during the culture days, as shown in Fig. 1B. The isolated cells exhibit the desired specific phenotype markers CYP19 and CYP17A1 to verify the isolated cells, respectively (Fig. 1C). In addition, we conducted flow cytometry for each cell-specific marker to quantitatively assess the purity of the isolated cells (Fig. 1D). The flow cytometric analysis showed that 98.3% of the cells stained positive for CYP19 (considered as GCs obtained from puncturing the ovaries) and 96.8% of the cells stained positive for CYP17A1 (considered as TCs from punctured ovarian tissue).

### Preparation of GTBs via electrostatic extrusion method

**Fig. 2.**
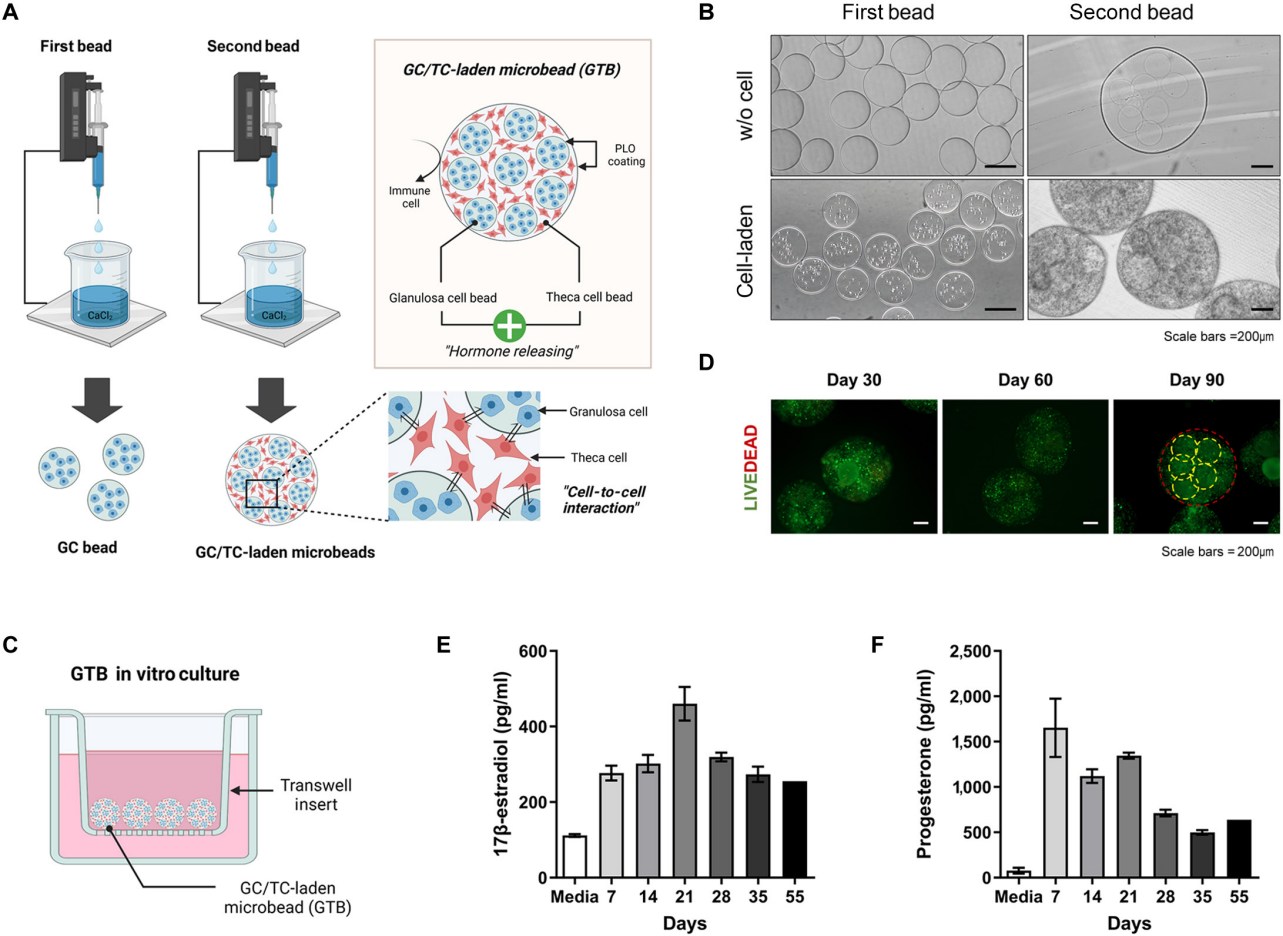
Fabrication of GC/TC-laden microbeads (GTBs) capable of producing ovarian hormones. (A) The electrostatic extrusion method was used to produce GTB, as shown in the scheme. Briefly, the first bead (GC bead), composed of granulosa cells, is constructed, and subsequently, the second bead (GTB) is fabricated by mixing the previously created GC beads with theca cells. (B) Images of the first bead and the second bead with or without cells. Scale bars, 200 μm. (C) In vitro culture of GTBs using the Transwell system for a duration of 90 days. (D) Live/dead images of GTBs on days 30, 60, and 90. Scale bars, 200 μm. (E and F) Measurement of ovarian sex hormone (17β-estradiol and progesterone) level from GTB spent medium. Data are means ± SEM.

The ovary is an organ having a small and oval-like shape and contains ovarian follicles inside. To construct ovarian structures, we prepared alginate beads in 2 steps via the electrostatic extrusion method (Fig. 2A). The large microbeads are produced with small beads to fabricate biomimetic artificial structures. Specifically, the small beads were around 200 μm produced under 9 kV with a 28-gauge needle with 1.5% alginate containing GCs (Fig. 2B). The beads produced secondly with small beads were 800 to 1,000 μm in size and contained 8 to 10 beads, using a 20-gauge needle with 1.5% alginate containing TCs. During the 90-day in vitro culture, the GTBs exhibited an “alive” state, consistent with the duration applied in the in vivo experiment (Fig. 2C and D). Next, we confirmed the sustained release of ovarian sex steroid hormones (E_2_ and P_4_) from the fabricated GTBs over a duration corresponding to 2 menstrual cycles in humans (Fig. 2E and F).

### Application of GTBs in a menopausal rat model

**Fig. 3.**
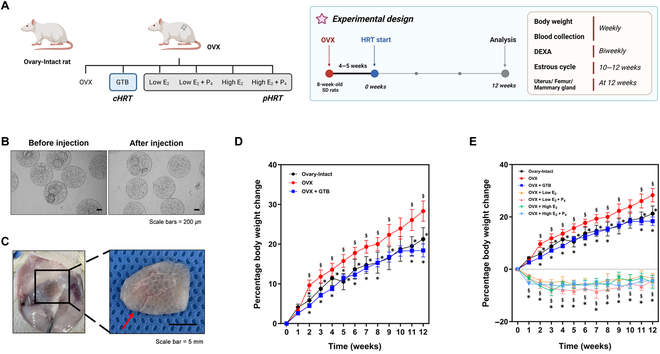
Application of GTBs in a menopause rat model. (A) Experimental groups and design (cHRT, cell-based HRT; pHRT, pharmaceutical HRT). (B) Images of GTB morphology before and after injection. Scale bars, 200 μm. (C) Images of injected GTBs retrieved at 12 weeks after injection. Red arrow, blood vessel. Scale bar, 5 mm. Body weight changes over 12 weeks in OVX rats administered with either GTB (D) or pharmaceutical hormone (E), in comparison to ovary-intact and OVX rats. Data are means ± SEM. ^§^*P* < 0.05 versus Ovary-Intact. *P < 0.05 versus OVX.

To investigate the effects of GTB as HRT, we induced a menopausal model by bilaterally removing the ovaries of 8-week-old female rats, leading to impaired ovarian hormone production. HRT was initiated at approximately 4 to 5 weeks after OVX when ovarian hormone levels reached a baseline [28]. As shown in Fig. 3A, the experimental groups are constituted as follows: Ovary-Intact, OVX, GTB-injected OVX, and pharmaceutical hormone-treated OVX rats. Based on the dose of E_2_ and the presence of P_4_, pharmaceutical hormone-treated rats were divided into 4 groups. Body weight measurement and blood collection were conducted weekly, and DEXA scans were performed biweekly for 12 weeks. In addition, we checked the estrous cycle daily during the last 2 weeks. The uterus, femur, and mammary gland were collected at 12 weeks after the HRT. To ensure that the shear stress can affect the microbeads during injection, the GTBs confirmed no significant morphological changes, distortion, or blockage to the syringe (Fig. 3B). After 12 weeks following the injection, shown in Fig. 3C, the GTBs remained securely in their position, and there was clear evidence of well-developed blood vessels in the surrounding area (red arrow in the inserted photograph, Fig. 3C). This result indicates favorable and stable outcomes related to the treatment or procedure being assessed.

As shown in Fig. 3D and E, the OVX rats have changed 28.38% of their body weight after 12 weeks. The GTB-injected rats reduced the increasing rates 2 weeks after injecting GTBs, which recovered similar levels to ovary-intact rats. In the case of pharmaceutical HRT, including the high and low doses of E_2_ (with or without P_4_), the body weight was decreased immediately after treatment and then maintained in this study. It appears that estrogen is directly administered into the body, exerting immediate effects in the pharmaceutical HRT groups. After 12 weeks, the OVX rats that received pharmaceutical HRT changed their body weight to −3.45% for low E_2_, −4.49% for low E_2_ + P_4_, −4.94% for high E_2_, and −4.59% for high E_2_ + P_4_ treatment, while the body weight of the GTB-injected rats was, on average, 18.4% that of ovary-intact rats. These indicated that treatment of the GTB constructs prevented weight gain after OVX, such as accumulation of fat caused by sex hormone deficit.

### Recovery of hormone production

**Fig. 4.**
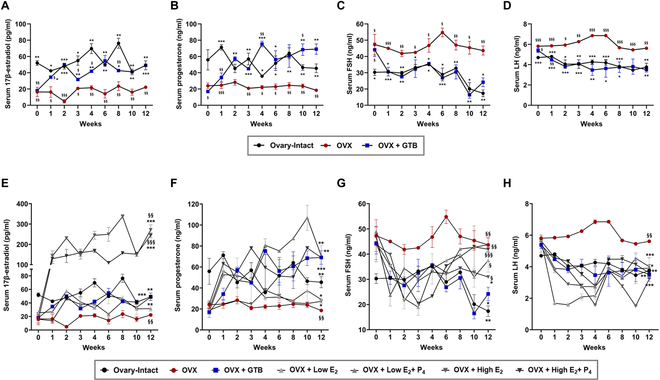
Recovery of ovarian hormone production and negative feedback loop in GTB-treated OVX rats. Analysis of serum concentrations of 17β-estradiol (A and E) and progesterone (B and F), FSH (C and G), and LH (D and H). A to D represent OVX rats treated with GTB. E to H show the results comprehensively, including those treated with pharmaceutical hormone, and statistical significance was indicated only at week 12. The significance of all pharmaceutical hormone-treated OVX groups over the 12 weeks is noted in Figs. S1 and S2. Data are means ± SEM. ^§^*P* < 0.05, ^§§^*P* < 0.01, and ^§§§^*P* < 0.001 versus Ovary-Intact. *P < 0.05, **P < 0.01, and ***P < 0.001 versus OVX. (For a clearer view of the pharmaceutical group’s results, please refer to Figs. S1 and S2.)

The serum levels of E_2_ and P_4_ in OVX rats exhibited a notable decrease from the onset of HRT at the fourth week after OVX compared to ovary-intact rats (Fig. 4A and B). The ovarian sex hormone (E_2_ and P_4_) levels that were reduced due to ovariectomy were restored through the injection of GTBs until the 12th week, reaching levels comparable to the ovary-intact rats. The GTB-injected rats exhibited a significant decline in serum levels of both FSH and LH compared to OVX rats, showing similar levels observed in ovary-intact rats (Fig. 4C and D). In pharmaceutical HRT, regardless of the delivery of P_4_, the serum E_2_ levels significantly increased in low-dose E_2_ groups compared to OVX at the 2 weeks after hormone delivery, but these levels tended to remain lower than those observed in ovary-intact rats over the 12 weeks (Fig. 4E and Fig. S1A). However, the high-dose E_2_ groups showed significantly elevated E_2_ levels, exceeding those measured in the ovary-intact rats (Fig. 4E and Fig. S1B). The serum levels of P_4_ were similar to or higher than the ovary-intact rats in the pharmaceutical HRT combined with P_4_, while they were similar to the OVX rats in the pharmaceutical HRT with E_2_-only groups (Fig. 4F and Fig. S1C and D).

While there was a suppression effect of FSH production initially in all pharmaceutical hormone-treated rats, it was observed that the inhibitory effect of FSH decreased at the final 12 weeks, particularly in E_2_-only-treated OVX groups (Fig. 4G and Fig. S2A and B). Similar to the serum FSH level, the serum level of LH exhibited a transient decrease in the early period after the initiation of pharmaceutical HRT, but these inhibitory effects were maintained in all rats treated with the pharmaceutical hormone compared to OVX rats over the 12 weeks (Fig. 4H and Fig. S2C and D). Through the recovery of ovarian hormone levels, we confirmed the GTBs, when applied in vivo, also function as hormone producers like the natural ovary.

### Revert endometrium and estrous cycle after injection of GTB

**Fig. 5.**
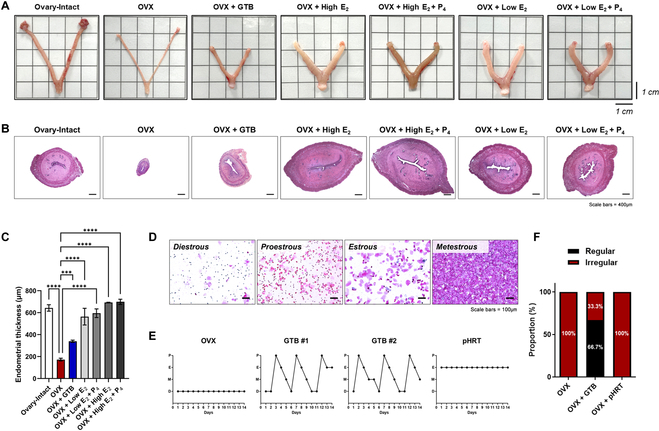
Endometrium regeneration and estrous cycle recovery by GTB. (A) Images of uterine gross in ovary-intact and OVX treated with either GTB or pharmaceutical hormone. Scale bars, 1 cm. (B) Representative H&E histology showing cross-sections of the uterus. Scale bars, 400 μm. (C) Quantitative analysis of endometrium thickness through ImageJ software. (D) H&E stain images for each stage of the estrous cycle. Scale bars, 100 μm. (E) Representative estrous cycling patterns from OVX, OVX + GTB, and OVX + pharmaceutical HRT (pHRT) groups for 14 days (P, Proestrous; E, Estrous; M, Metestrous; D, Diestrous). (F) The ratio of rats exhibiting regular (black) or irregular (red) estrous cycle in OVX, OVX + GTB, and OVX + pHRT groups. Data are means ± SEM. ***P < 0.001 and ****P < 0.0001 compared to OVX.

Ovarian hormone deficiency resulting from OVX rats is associated with a decrease in the thickness of the uterine endometrium. Therefore, we examined the uterus gross and endometrium thickness at 12 weeks after the initiation of the HRT. As shown in Fig. 5A, whereas the OVX rats had thin uterine endometrium compared to ovary-intact rats, OVX rats treated with GTB or pharmaceutical hormone showed a restoration of uterine morphology. Next, we analyzed quantitative measurements of endometrial thickness by H&E staining (Fig. 5B and C). The GTB-injected rats showed a significantly increased endometrium thickness compared to OVX rats, indicating an endometrium regeneration through hormonal release from the injected GTBs.

Moreover, the estrous cycle involves complex interactions among various hormones to regulate the diverse stages of the reproductive process. The estrous cycle consists of 4 stages: diestrous, proestrous, estrous, and metestrous (Fig. 5D). In the OVX rats, a prolonged diestrous stage was shown, while in pharmaceutical hormone-treated OVX rats, a sustained proestrous or estrous stage was noted. However, the GTB-injected rats showed a normal estrous cycle in 66.7% of individuals (Fig. 5E and F). Figure S3 shows the estrous cycle for all 9 individuals treated with GTB during the last 2 weeks.

### Prevention of osteoporosis

**Fig. 6.**
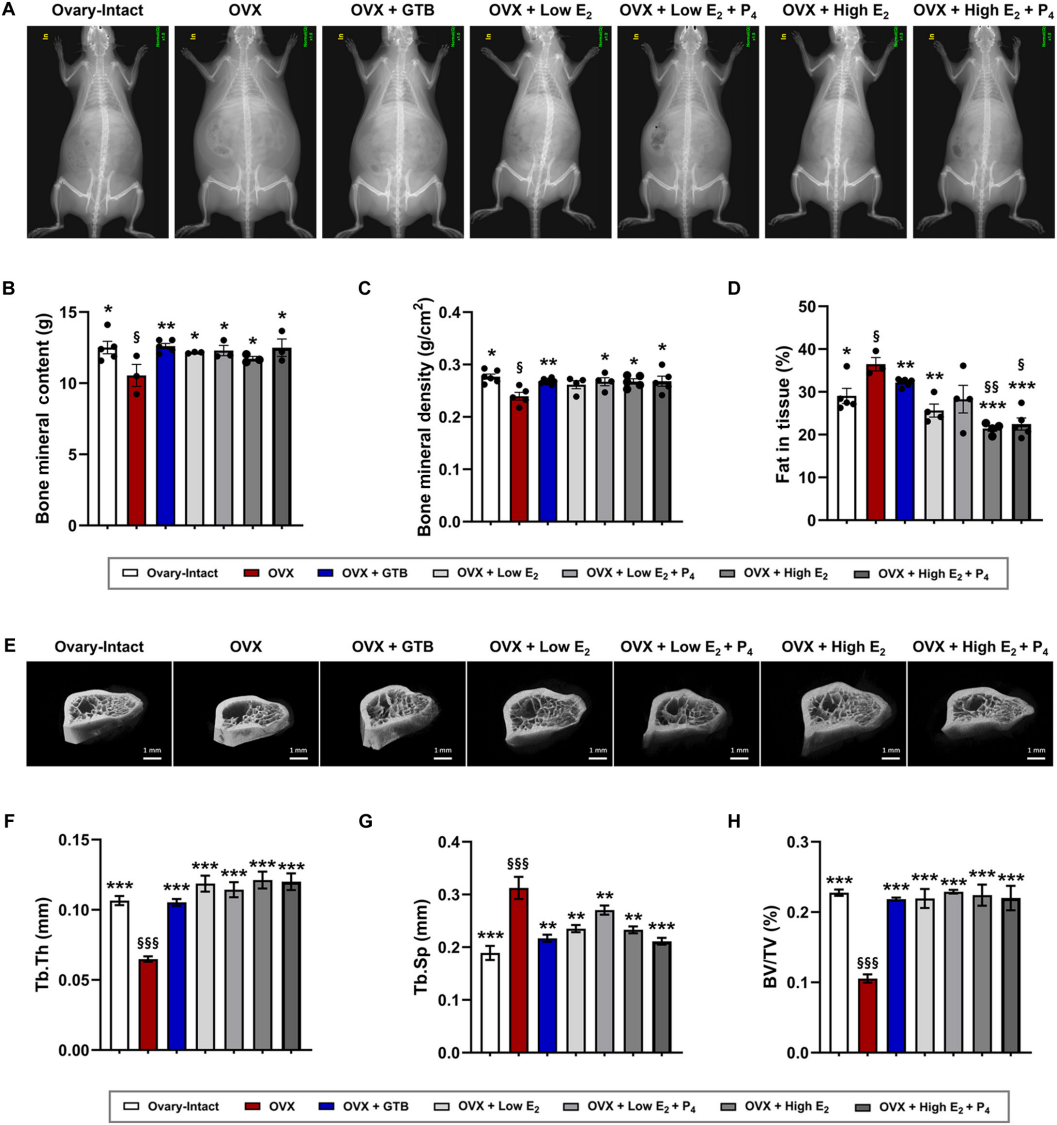
Protection against osteoporosis risk using GTB treatment. (A) Representative images obtained from dual-energy x-ray absorptiometry (DEXA) and quantitative analysis of DEXA images, including bone mineral content (B), bone mineral density (C), and fat in tissue (D). (E) Representative 3D images of femoral architecture and porosity using μCT with quantitative analysis, including trabecular thickness (Tb.Th) (F), trabecular separation (Tb.Sp) (G), and bone volume/total volume (BV/TV) (H) in GTB or pharmaceutical hormone-treated OVX rats compared to ovary-intact and OVX rats. Scale bars, 1 mm. Data are means ± SEM. ^§^*P* < 0.05, ^§§^ *P* < 0.01, and ^§§§^ *P* < 0.001 versus Ovary-Intact. *P < 0.05, **P < 0.01, and ***P < 0.001 versus OVX.

After 12 weeks after treatment, the OVX rats showed lower BMC and BMD and higher fat in tissue than ovary-intact rats (Fig. 6A to D). Similar to pharmaceutical hormone-treated groups, the GTB-treated group showed a recovery tendency of all parameters with significant differences compared to OVX rats. When observed bi-weekly, BMC rescue was seen at 4 weeks, BMD at 8 weeks, and fat in tissue at 2 weeks after GTB injection (Figs. S4 to S6). The result means GTB treatment has adequately acted as HRT and efficacy of prevention bone loss. In the pharmaceutical HRT groups, BMC and BMD were maintained at levels comparable to the ovary-intact rats (Fig. 6A to C and Figs. S4 and S5). However, in particular, the high-dose E_2_-administrated rats showed a considerable reduction of fat in tissue compared to ovary-intact rats, whose result is similar to body weight change (Figs. 3E and 6D and Fig. S6).

The μCT scans showed obvious differences in trabecular bone architectures, such as porosity (Fig. 6E). OVX rats also showed a decline in Tb.Th and an enhancement in Tb.Sp (Fig. 6F and G). The Tb.Th and the Tb.Sp of OVX rats were 60.86% and 165.24% compared to ovary-intact rats. The BV/TV was 10.54%, as shown in Fig. 6H. GTB and pharmaceutical hormone-treated OVX rats recovered all parameters in the range of ovary-intact rats. The GTB treatment restored 98.76% of Tb.Th and 114.74% of Tb.Sp (% of ovary-intact rats). This observation showed the restoration of bone architecture and prevention of progressive osteoporosis through our GTB treatment as much as pharmaceutical HRT.

### Potential reduction of breast cancer risk

**Fig. 7.**
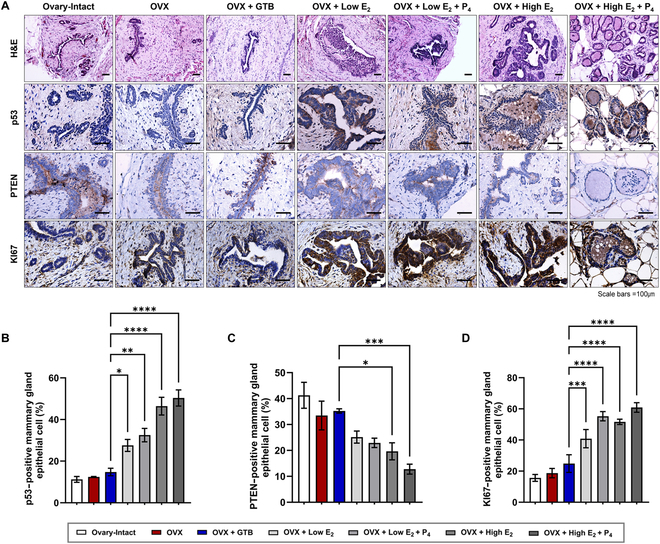
Attenuation of mammary gland hyperplasia by GTB-based HRT. (A) Representative H&E histology showing cross-sections of the mammary gland at 12 weeks after HRT. Representative immunohistochemistry images of the expression of p53 and PTEN as cancer markers and the expression of KI67 as a proliferation marker. Scale bars, 100 μm. The quantification of p53 (B), PTEN (C), and KI67 (D) positive mammary gland epithelial cell ratio in pharmaceutical hormone-treated OVX rats compared to GTB-injected rats. Data are means ± SEM. *P < 0.05, **P < 0.01, ***P < 0.001, and ****P < 0.0001 versus OVX + GTB.

To verify one of the primary adverse effects of HRT, specifically the risk of developing breast cancer, we examined the morphology of the mammary gland through H&E stain. As shown in Fig. 7A, mammary hyperplasia, such as the presence of cells within the mammary gland lumen and multilayered epithelium, was observed in all pharmaceutical HRT groups. Furthermore, we observed pseudo-lactational hyperplasia in high-dose E_2_ groups. In contrast, the GTB-injected OVX rats did not exhibit characteristics of hyperplasia and showed a morphology like the ovary-intact rats (Fig. 7A).

GTB-injected rats did not exhibit significant differences in the expression of KI67, p53, and PTEN compared to both ovary-intact and OVX rats (Fig. 7). However, all pharmaceutical HRT groups expressed a notable rise in the number of p53-positive mammary gland epithelial cells (Fig. 7A and B), whereas there was a decrease in the number of PTEN-positive mammary gland epithelial cells compared to the GTB-injected OVX rats (Fig. 7A and C). In our investigation, we noted a considerable elevation in KI67 expression across all pharmaceutical HRT groups when compared to OVX rats injected with GTB (Fig. 7A and D). Moreover, there was a tendency for a higher increase in groups with the addition of P_4_ or the high-dose E_2_-treated group.

## Discussion

Our study demonstrates the efficacy of an injectable cell-based HRT using isolated ovarian cell-embedded hydrogel beads, which eliminates the need for transplantation. The GTB, as shown in this study, can be prepared using the electrostatic extrusion method and biomimetic strategy of the ovarian structure to embody natural communication between brain-reproductive organs. We conducted the injection of our hydrogel constructs after OVX, in which the rats recovered hormonal activity and prevented menopause-related symptoms.

We successfully isolated primary endocrine cells from E_2_-primed rats and cultured the cells to prepare GTBs for our injectable cell-based HRT (Figs. 1 and 2). Since the in vitro 2-dimensional culture system cannot reflect the original environment for GCs and TCs, the cells are cultured in the growth medium in the presence of hormones [29,30]. It leads to maintaining the function and morphology of GCs and TCs in vitro. As a result, we demonstrated that the isolated cells maintained their phenotype after culturing in vitro. The original ovary is divided into 2 main parts: the inner medulla is soft, and the outer cortex has rigid properties, depending on the structure and components of the extracellular matrix (ECM) [31,32]. Therefore, we induced a mechanical gradient of GTBs by crosslinking different calcium solutions under each preparation step.

Production of E_2_ and P_4_ was measured in in vitro GTB culture for 55 days. It is known that humans have an average menstrual cycle of 28 days [33]. Steroidogenesis can occur by interacting with GCs and TCs even under in vitro conditions. The previous study confirmed hormone production co-culturing of these endocrine cells, and the layered encapsulation can lead to efficient steroidogenesis in vitro [13]. Our results from hormone measurement in in vitro culture were similar, which means our GTBs act as hormone producers on behalf of the original ovaries. It indicated not only that GCs and TCs in the GTB are close enough to interact but also that they can communicate with each other in the alginate hydrogel constructs.

We injected GTBs into the rat subcutis after mixing them with 5% HA after washing with distilled water and HBSS to determine whether GTBs can replace ovaries as a hormone producer. We used HA as a carrier of microbeads because it has the proper viscosity, does not get stuck in a bottleneck when applying the injection, and is biocompatible with host tissue after injection. In addition, HA is a well-known angiogenesis inducer identified as a key component of the natural ECM [34,35]. After injecting GTBs, the body weight had been changed during the observation period. Weight gain after the peri- and postmenopausal period is well-known, and it is widely recognized that reduced ovarian function causes a hormonal deficit, leading to the accumulation of fat in the body [36]. Estrogen, produced by the ovary, is involved in regulating fat deposition, leading to weight loss when HRT is administered [37]. As shown in Fig. 3D and E, our results were similar to those of previous studies; the body weight increases typically in OVX rats.

Hormone production is one of the paramount roles of the ovary as a reproductive organ in females. After menopause or losing ovarian function, the patients suffer from such side effect symptoms resulting from an imbalance of hormones [38]. The HRT is a treatment for compensating their hormones such as estrogen and progesterone. As mentioned above, our GTBs can produce the 2 major ovarian hormones in in vitro environments. We conducted experiments to react the GTBs as producers in an in vivo environment like the ovary. It was observed that ovarian sex hormone levels recovered after injection of GTBs (Fig. 4A and B). Both serum levels of E_2_ and P_4_ in the ovary-intact and GTB-injected rats displayed hormone level fluctuations. The observed variations can be linked to the influence of diverse estrous cycles at the time of blood collection, and these fluctuations are within the normal range [39]. The disruption of the HPO axis, essential for maintaining the female endocrine function, occurs in postmenopausal women due to reduced ovarian hormone production. This disruption fails to inhibit gonadotropin secretion, resulting in elevated FSH and LH levels [40]. The increased serum levels of FSH and LH in OVX rats are suppressed by the ovarian hormones released from GTBs (Fig. 4). However, serum LH levels in rats treated with pharmaceutical HRT were comparable to those observed in the ovary-intact rats, while the inhibition of FSH secretion through a negative feedback loop was slightly insufficient. Coupling with the previous results of recovery of ovarian sex hormone level in GTB-injected rats similar to ovary-intact rats, this indicates that the injected GTBs induced a balanced HPO axis through the normal action of the negative feedback system, leading to the restoration of endocrine function.

The uterine endometrium is a primary target tissue for estrogen [41]. Estrogen promotes the proliferation of the uterine endometrium, increasing the thickness of the uterus, and progesterone balances the action of estrogen, supporting the proper development and maintenance of the uterine endometrium [42]. Although the endometrial thickness in GTB-injected rats has not fully recovered compared to the ovary-intact rats, we concluded that the current dose of GTB is sufficient for regenerating the thinned endometrium caused by menopause because of a significant increase in endometrial thickness in the GTB group compared to the OVX group. The pharmaceutical HRT groups, especially in the high-dose E_2_-treated group, exhibited a further increase in endometrial thickness, and even in the low-dose E_2_ group, a significant increase was observed compared to OVX rats. This indicates that both GTB and pharmaceutical hormone injection methods release a sufficient amount of hormones to regenerate the thinned endometrium, a symptom commonly associated with menopause. The recovery of the estrous cycle implies a stabilized feedback system among ovarian hormones, GnRH, and pituitary hormones. In this study, we observed the recovery of the estrous cycle in GTB-injected rats (Fig. 5F). Furthermore, it was confirmed that the levels of the anterior pituitary hormones FSH and LH also recovered to normal levels in the GTB group (Fig. 4C and D). Taken together, GTB-injected rats had a balanced HPO axis, highlighting the importance of maintaining female endocrine function through the restoration of the estrous cycle, along with the regeneration of the endometrium. The observed distinctions between GTB-injected and pharmaceutical HRT-treated groups highlight potential physiological differences between the 2 HRT approaches. Cell-based HRT, unlike its pharmaceutical counterpart, can generate ovarian hormones within the intact feedback mechanism of the HPO axis. In contrast, pharmaceutical HRT delivers hormones exogenously, lacking the endogenous hormone production regulated by the HPO axis. This fundamental difference may contribute to the disparity in potential side effects between the 2 methods. Due to its regulatory nature, cell-based HRT may prevent hormonal excess and, consequently, could mitigate related side effects more effectively than pharmaceutical HRT.

Osteoporosis is representative of menopausal symptoms, marked by the gradual decline in bone mass and structural deterioration [43]. These alternations contribute to elevated risks of bone fractures and structural defects. In this study, we tracked the changes in BMD, BMC, and fat in tissue during the experimental period. The reduction of Tb.Th is one major sign of osteoporosis progress, and the thin trabecular bone leads to easier bone fracture [44]. Furthermore, the Tb.Sp increases with thinner trabecular bone, generating greater cavities in the bone.

Mammary hyperplasia is a precancerous condition that is characterized by the overgrowth of cells in lobules and ducts [45]. Due to the potential risk of hyperplasia processing breast cancer, we investigated the expression of cancer markers (p53 and PTEN) and proliferation markers (KI67) by immunohistochemistry. The expression of p53 shows accumulation in proliferative lesions, whereas, conversely, PTEN is usually expressed in normal glandular epithelial cells [46,47]. Based on this, it is known that elevated p53 and declined PTEN expression are associated with an increased risk of breast cancer. These trends were further heightened in the pharmaceutical HRT with the addition of P_4_. This aligns with the fact that the pharmaceutical HRT, which is a combination of E_2_ and P_4_, increases the probability of breast cancer [48]. KI67, a marker commonly associated with proliferating cells, is frequently observed to be overexpressed in various breast cancer cases [49]. Indeed, it has been known that a significant up-regulation of KI67 was detected in both non-atypical and atypical hyperplasia [45,49]. In our study, we observed that the pharmaceutical HRT groups exhibited hyperplasia and an elevated risk of breast cancer, whereas the GTB group did not show these adverse effects (Fig. 7). As shown in Fig. 4, the estrogen and progesterone directly administered in the pharmaceutical HRT group did not reflect the physiological regulation of hormones within the body, leading to the excessive proliferation of mammary gland epithelial cells. In contrast, in the cell-based HRT group, excessive proliferation of mammary gland epithelial cells was prevented by maintaining the physiological balance of hormone regulation as an artificial ovary. It is also assumed that the response to hormones in each organ where estrogen and progesterone act differs between endogenous and exogenous hormones. Unlike premenopausal women where endogenous hormones are active, the increased incidence of breast cancer in postmenopausal women undergoing exogenous hormone therapy (pharmaceutical HRT) suggests this difference clinically. These results suggest that cell-based HRT using GTB can serve as an alternative HRT capable of alleviating the breast cancer risk associated with conventional HRT.

In summary, our GTBs not only prove the effectiveness of hormone therapy but also suggest the feasibility of a non-invasive approach in cell-based HRT. The injectable treatment is easy to use, and the beads can simulate the feedback system of real ovaries, enabling hormone production and maintenance compared to conventional HRT. We extended this approach by applying injectable hydrogels to cell-based HRT in our research, demonstrating the GTBs as hormone providers that communicate with the brain to balance normal hormone levels. Our findings emphasize the effectiveness of hormone therapy through the easily injectable ovarian mimic beads. The injectable artificial ovary for HRT introduces a novel, non-invasive method for cell-based HRT, with potential for adaptation across a variety of cell-based treatments. Our method suggests that cell-based hormone therapy may mitigate the risk of breast cancer, a major side effect associated with pharmaceutical HRT. Furthermore, the long-term implications and potential for personalized treatment make this cell-based approach a compelling avenue for future research and clinical applications. As we continue to unravel the intricacies of this innovative HRT, it holds promise not only in addressing menopausal symptoms but also in minimizing adverse effects, paving the way for a more sustainable and patient-friendly treatment option.

## Data Availability

All data that support the findings of this study are included within the article (and any supplementary files).
