# Supplementary material for: Injectable Biomimetic Hydrogel Constructs for Cell-Based Menopausal Hormone Therapy with Reduced Breast Cancer Potential
Source: Biomater Res. 2024 Aug 9;28:0054. doi: 10.34133/bmr.0054 (PMC11310713; doi:10.34133/bmr.0054)
Supplement: Supplementary 1 — Figs. S1 to S6 [file bmr.0054.f1.zip › BMR_LicenseToPublish2024_JR Lee.pdf]

Biomaterials Research
A SCIENCE PARTNER JOURNAL

License to Publish

I. Requirement of Acceptance:

The following Grant of License ("License") must be signed and returned to the Korean Society for Biomaterials ("KSBM") before a manuscript can be accepted for publication in *Biomaterials Research*.

By signing this License, you represent and warrant that you have the authority and all necessary rights to execute this License. For example, if your institution places limitations on publishing agreements or asserts its own right to distribute or provide access to the works of its faculty, you must obtain a waiver from your institution that releases you from such restrictions so that you can sign this License. After publication of your manuscript in *Biomaterials Research*, your institution may exercise all rights to use the Work as are retained by Authors in section III of this License.

If the copyright in the contribution is owned by your employer, your employer or an authorized representative must sign this form.

Should KSBM decide not to publish your manuscript, upon final written notification to you that KSBM will not publish your manuscript, this License shall be null and void. KSBM retains complete discretion as to whether to publish any manuscript, and in what form.

II. Rights of Publication

In consideration of publication in *Biomaterials Research* of the manuscript currently titled Injectable biomimetic hydrogel constructs for cell-based menopausal hormone therapy with reduced breast cancer potential (the "Work") and authored by Jung Ryeol Lee ("You"),

You hereby grant to KSBM the sole and exclusive, irrevocable right to publish, reproduce, distribute, transmit, display, store, translate, create derivative works from and otherwise use the Work, for any purpose, including commercial purpose, in any form, manner, format, or medium, whether now known or hereafter developed, throughout the world and in any language, for the entire duration of any such right and any renewal or extension thereof and to permit/sublicense others to do any or all of the foregoing as well.

With regard to associated supplemental materials, data, audio and/or video files that You have submitted for publication with Your manuscript, You hereby grant to KSBM

the non-exclusive right to publish, reproduce, distribute, transmit, display, store, translate, create derivative works from and otherwise use these supplemental materials in any form, manner, format, or medium, whether now known or hereafter developed, throughout the world and in any language, for the entire duration of any such right and any renewal or extension thereof and to permit/sublicense others to do any or all of the foregoing as well.

You retain copyright, subject to the rights You grant to KSBM above, and all rights not expressly granted in this License. No rights in patents or trademarks or other intellectual property rights other than as described above are transferred to Partner in this License.

You also authorize KSBM, but KSBM undertakes no obligation to, at its own expense, enforce the rights granted under this license on Your behalf against third parties whom KSBM believes to be infringing the copyright in the Work.

III. Author Rights

Articles published in *Biomaterials Research* will be published under the Creative Commons Attribution 4.0 International Public License (CC BY) and may be subject to an Article Processing Charge ("APC"). Upon publication of the Work in *Biomaterials Research*, You and Your co-authors may make all uses of the article permitted by the CC BY license, subject to all conditions of that license.

IV. User Access and Rights

KSBM will make the final published version of the Work freely accessible online without barriers or embargo. Subject to acceptance of the Journal by Pubmed Central (PMC), upon publication KSBM will submit the final published version of the Work to PMC/UKPMC for immediate release. Use of the published version of the Work by Yourself and others shall be subject to the terms of the Creative Commons Attribution 4.0 International Public License (CC BY). This license allows users to copy, redistribute, remix, transform, and build upon the Work, in any format or medium, for any purpose including commercial purpose, on a perpetual basis provided they credit the Work and the authors. Users must explain any changes that were made from the original and may not suggest the authors endorse the use. The resultant work must be made available under the same terms, and must include a link to the CC BY 4.0 International License.

The full legal code of the Creative Commons Attribution 4.0 International Public License may be found at https://creativecommons.org/licenses/by/4.0/legalcode

V. Additional Author Representations

You warrant and represent that:

- the Work is original;
- all the facts contained therein are true and accurate;
- the Work does not contain any libelous statements;
- the Work has not been published elsewhere;
- the Work does not infringe upon any copyright, proprietary, or personal right of any third party.

You agree to indemnify, defend and hold harmless KSBM against any claims in respect of the warranties above.

IMPORTANT: If the Work contains any material that is owned or controlled by a third party, You must obtain permission for its use and submit written evidence of that permission to Your Editor and You must clearly acknowledge the source and copyright holder of that material within the text of the Work.

By signing this License, You warrant that You have the full power to enter into this License. This License shall remain in effect throughout the term of copyright in the Work and may not be revoked without the express written consent of both parties. This License shall be governed and construed, and any dispute arising hereunder resolved, in accordance with the laws of the District of Columbia, United States of America, without resort to the conflicts of laws principles thereof.

Jung Ryeol Lee

Author's Name (please print)

Author's Signature

For works created under U.S. Government Contract or as part of official duties as an employee of the U.S. Government, check the appropriate box and sign below instead of above:

☐ *This work was created under U.S. Government Contract*
Grant Number:___________

The AAAS recognizes the U.S. Government's non-exclusive rights to use the Work for non-commercial, governmental purposes where such rights are established in the grant or contract.

☐ *This work was written as part of Your official duties as an employee of the U.S. Government (and therefore the article is in the public domain).*

____
Author's Name (please print)

____
Author's Signature
