# Supplementary material for: Injectable Biomimetic Hydrogel Constructs for Cell-Based Menopausal Hormone Therapy with Reduced Breast Cancer Potential
Source: Biomater Res. 2024 Aug 9;28:0054. doi: 10.34133/bmr.0054 (PMC11310713; doi:10.34133/bmr.0054)
Supplement: Supplementary 1 — Figs. S1 to S6 [file bmr.0054.f1.zip › Supplemental Material.docx]

**Supplementary Information**

**Title**

Injectable biomimetic hydrogel constructs for cell-based menopausal hormone therapy with reduced breast cancer potential

**Authors**

Chungmo Yang^1,2†^, Heeseon Yang^1,3†^, Hyerim Kim^2^, Nanum Chung^1,3^, Jungwoo Shin^1,3^, Hyewon Min^1,3^, Kangwon Lee^4,5*^, Jung Ryeol Lee^1,3,6*^

**Affiliation**

1. Department of Obstetrics and Gynecology, Seoul National University Bundang Hospital, Seongnam 13620, Republic of Korea
2. Program in Nanoscience and Technology, Graduate School of Convergence Science and Technology, Seoul National University, Seoul 08826, Republic of Korea
3. Department of Translational Medicine, College of Medicine, Seoul National University, Seoul 03080, Republic of Korea
4. Department of Applied Bioengineering, Graduate School of Convergence Science and Technology, Seoul National University, Seoul 08826, Republic of Korea
5. Research Institute for Convergence Science, Seoul National University, Seoul 08826, Republic of Korea
6. Department of Obstetrics and Gynecology, Seoul National University College of Medicine, Seoul 03080, Republic of Korea

† These authors contributed equally to this work

*Co-corresponding authors


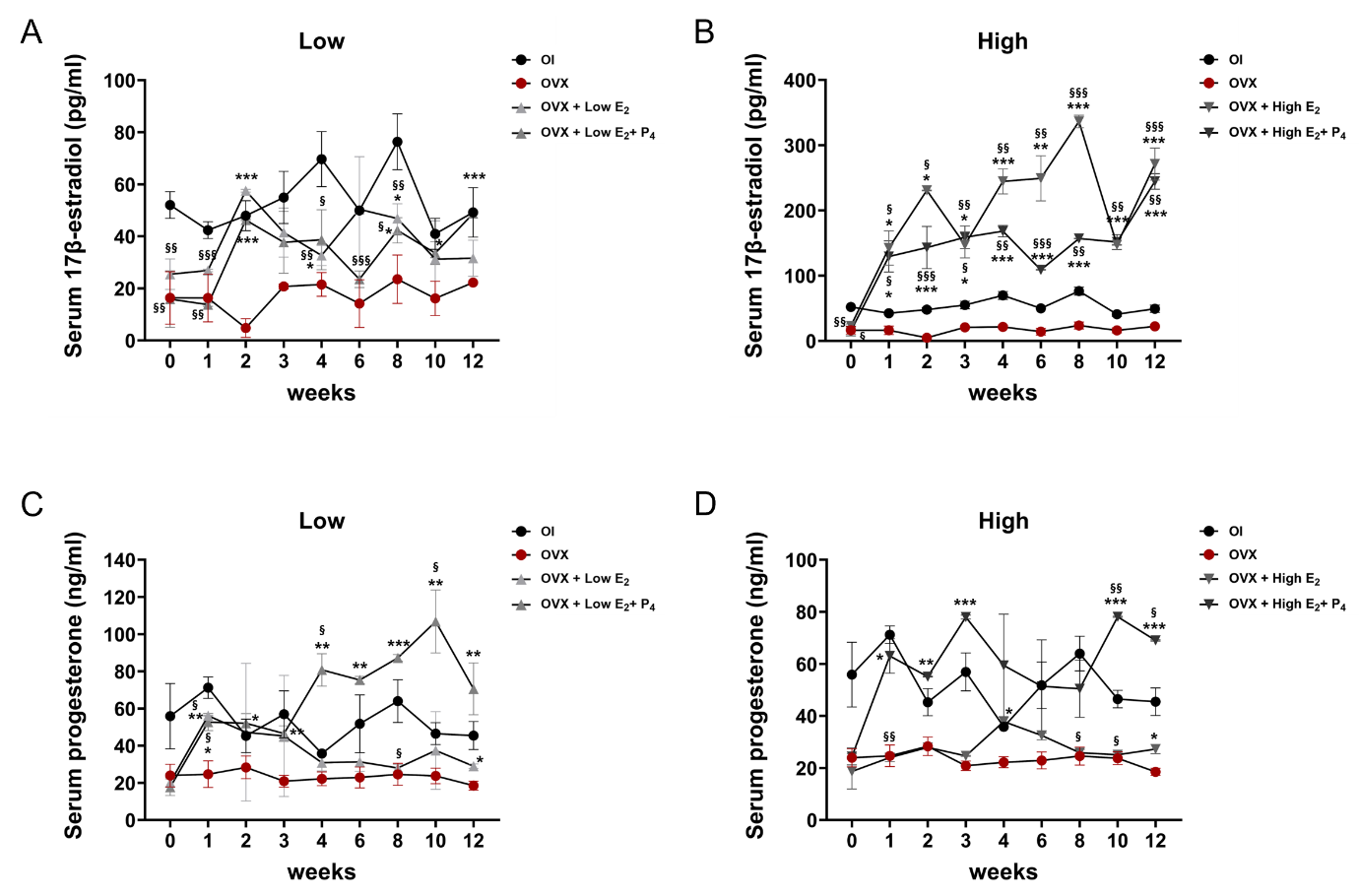
**Fig. S1.** Serum ovarian hormone level in pharmaceutical hormone-based HRT treated OVX rats. Analysis of serum concentrations of 17β-estradiol (**A**) and progesterone (**C**) in OVX rats treated with low-dose E_2_ group (OVX + Low E_2_ and OVX + Low E_2_ + P_4_) compared to ovary-intact (OI) and OVX rats. Analysis of serum concentrations of 17β-estradiol (**B**) and progesterone (**D**) in OVX rats treated with high-dose E_2_ group (OVX + High E_2_ and OVX + High E_2_ + P_4_) compared to OI and OVX rats. Data are means ± SEM. ^§§^ *P* < 0.01, and ^§§§^ *P* < 0.001 versus Ovary-Intact. **P* < 0.05, ***P* < 0.01, and ****P* < 0.001 versus OVX.

**Fig. S2.** Serum FSH and LH concentrations in pharmaceutical hormone-based HRT treated
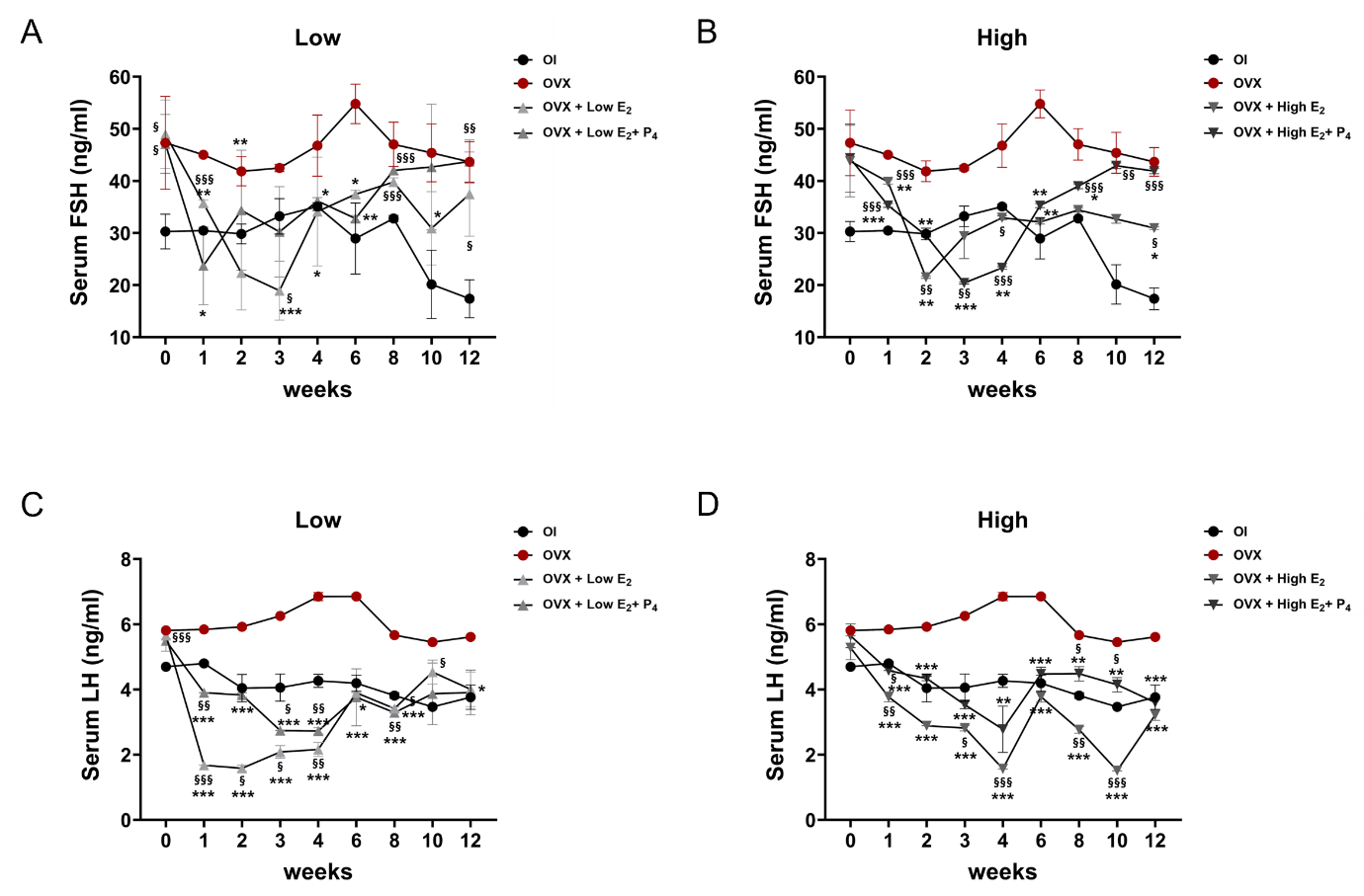
OVX rats. Analysis of serum concentrations of FSH (**A**) and LH (**C**) in OVX rats treated with low-dose E_2_ group (OVX + Low E_2_ and OVX + Low E_2_ + P_4_) compared to ovary-intact (OI) and OVX rats. Analysis of serum concentrations of FSH (**B**) and LH (**D**) in OVX rats treated with high-dose E_2_ group (OVX + High E_2_ and OVX + High E_2_ + P_4_) compared to OI and OVX rats. Data are means ± SEM. ^§§^ *P* < 0.01, and ^§§§^ *P* < 0.001 < 0.001 versus Ovary-Intact. **P* < 0.05, ***P* < 0.01, and ****P* < 0.001 versus OVX.


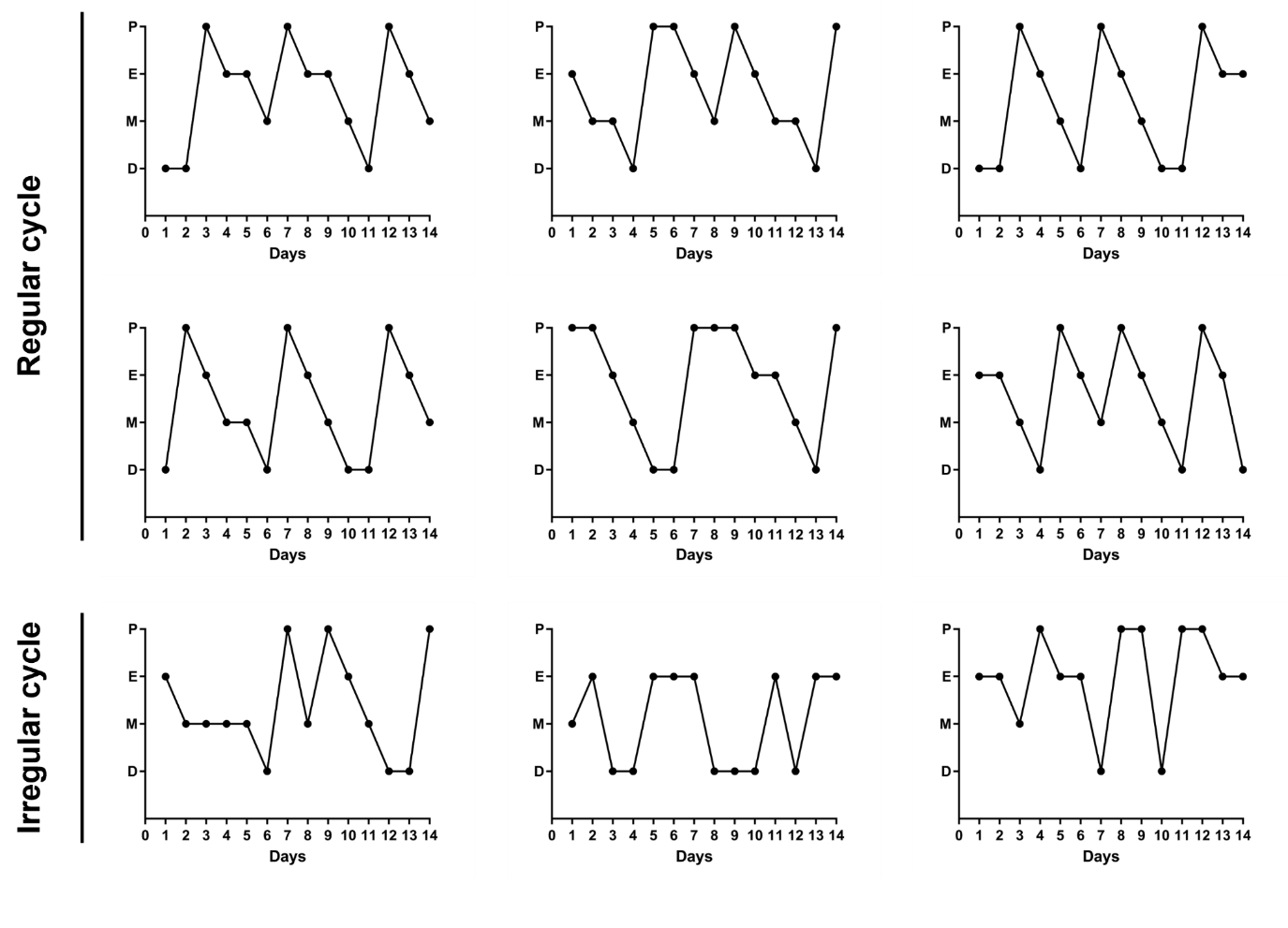
**Fig. S3.** Estrous cycling patterns in OVX treated with GTB. Analysis of estrous cycle for 2 weeks by vaginal cytology. Regular cycle; repeated every 4–5 days. Irregular cycle; prolonged estrous or disetrous cycle (each lasting more than 5 days) or cycles lasting more than 6 days. (P; Proestrous, E; Estrous, M; Metestrous, D; Diestrous). n=9.


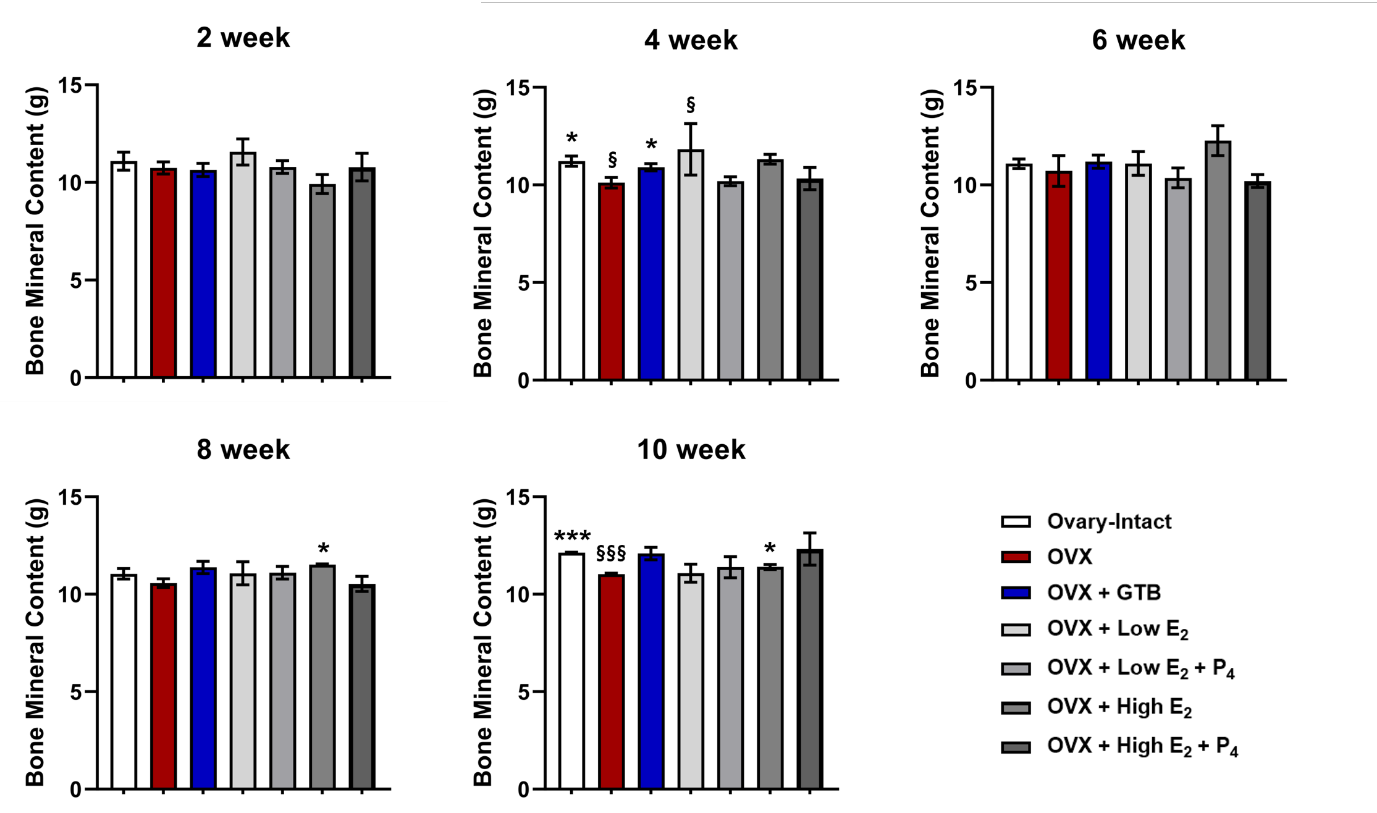
**Fig. S4.** Changes in bone mineral content (BMC) during HRT. Biweekly measurement of BMC by Dual-energy X-ray Absorptiometry (DEXA). Data are means ± SEM. ^§§^ *P* < 0.01, and ^§§§^ *P* < 0.001 versus Ovary-Intact. **P* < 0.05, ***P* < 0.01, and ****P* < 0.001 versus OVX.

**Fig. S5.** Changes in bone mineral density (BMD) during HRT. Biweekly measurement of BMD by Dual-energy X-ray Absorptiometry (DEXA). Data are means ± SEM. ^§§^ *P* < 0.01, and
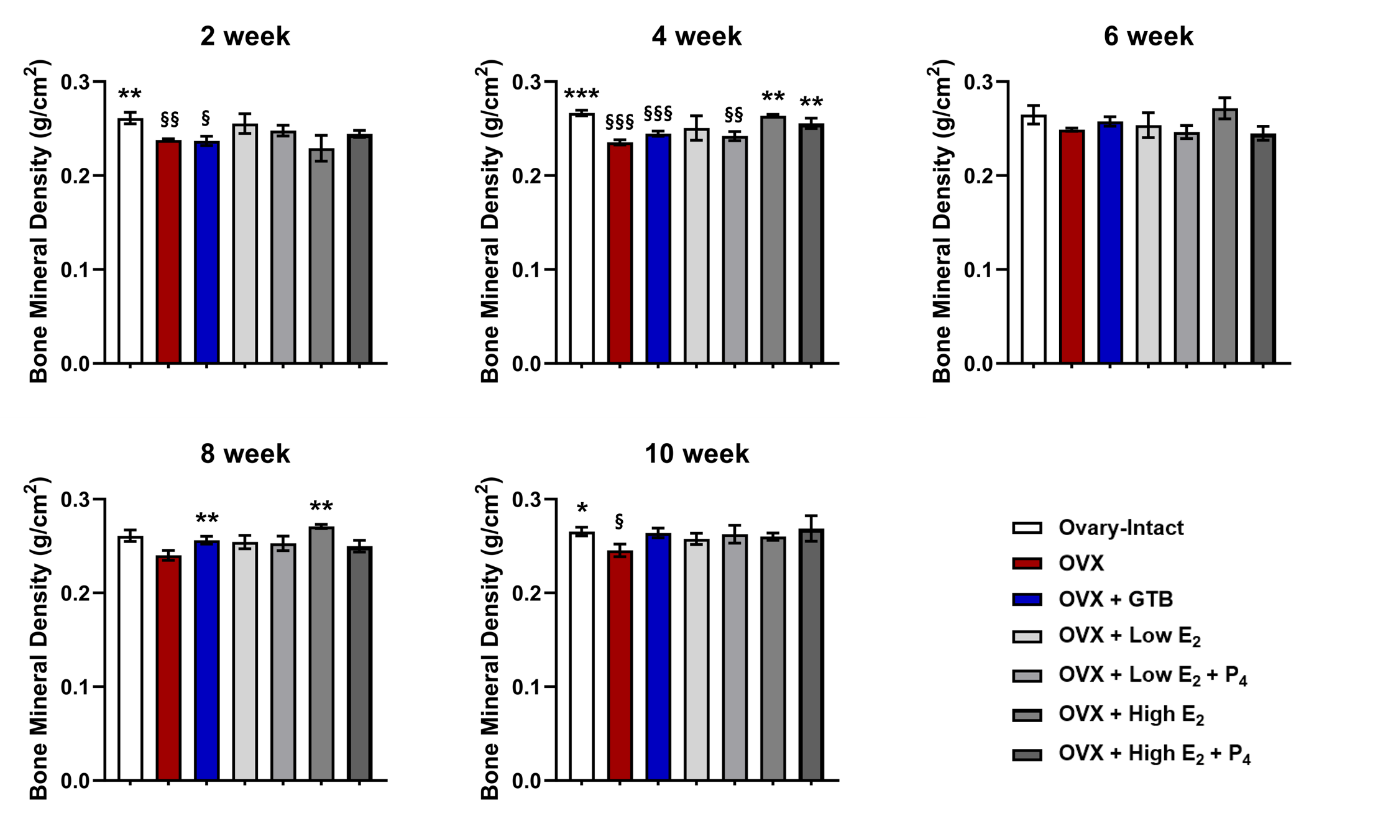
^§§§^ *P* < 0.001 versus Ovary-Intact. **P* < 0.05, ***P* < 0.01, and ****P* < 0.001 versus OVX.

**Fig. S6.** Changes in fat in tissue during HRT. Biweekly measurement of fat in tissue by
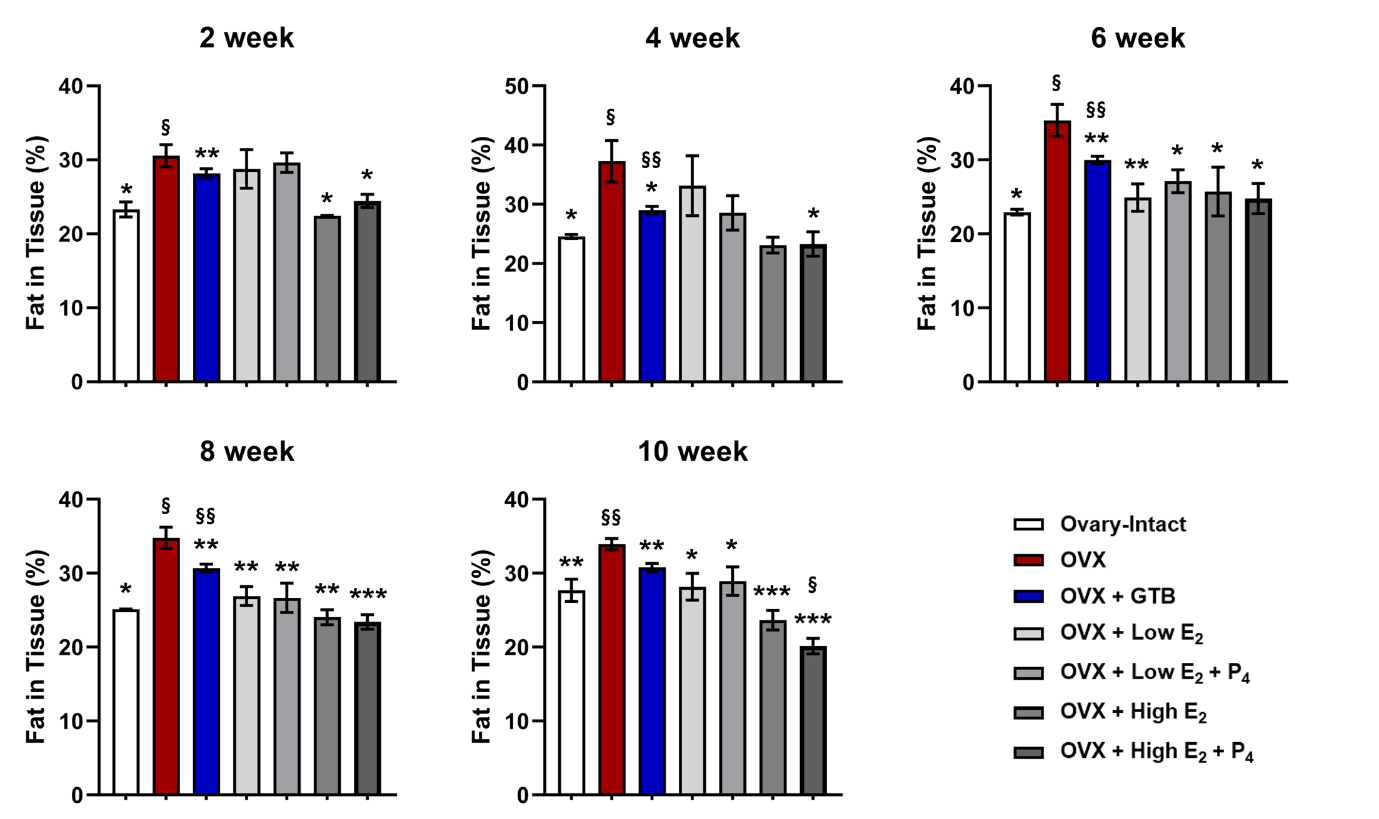
Dual-energy X-ray Absorptiometry (DEXA). Data are means ± SEM. ^§§^ *P* < 0.01, and ^§§§^ *P* < 0.001 versus Ovary-Intact. **P* < 0.05, ***P* < 0.01, and ****P* < 0.001 versus OVX.
