# Supplementary figures and images for: Injectable Biomimetic Hydrogel Constructs for Cell-Based Menopausal Hormone Therapy with Reduced Breast Cancer Potential
Source: Biomater Res. 2024 Aug 9;28:0054. doi: 10.34133/bmr.0054 (PMC11310713; doi:10.34133/bmr.0054)

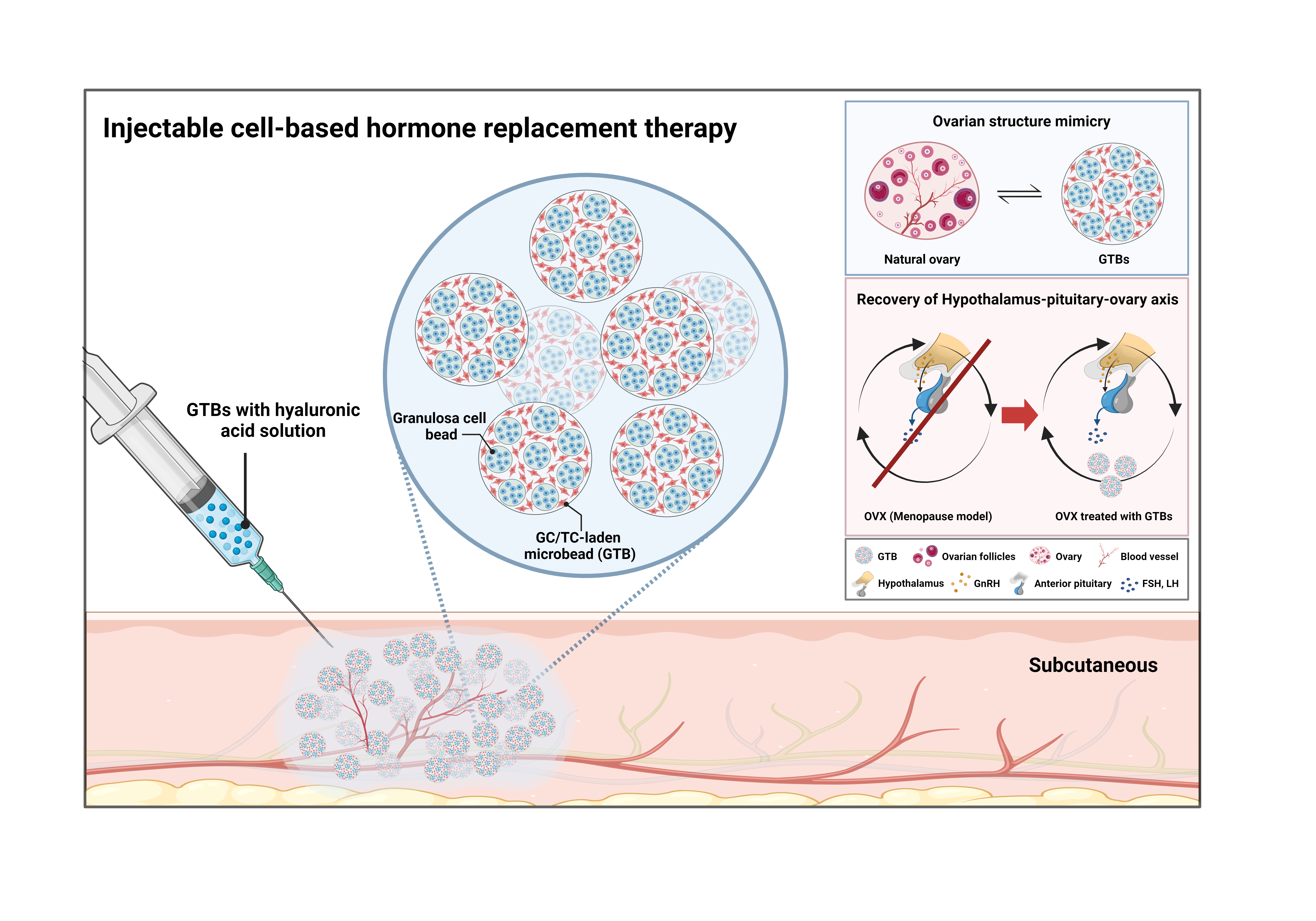

Supplement: Supplementary 1 — Figs. S1 to S6 [file bmr.0054.f1.zip › Graphical Abstract.png]
